# Systemic Inflammatory Markers for Predicting Overall Survival in Patients with Osteosarcoma: A Systematic Review and Meta-Analysis

**DOI:** 10.1155/2021/3456629

**Published:** 2021-10-21

**Authors:** Xiaotong Song, Hao Zhang, Fanxing Yin, Panpan Guo, Xiaocheng Yang, Jinzhu Liu, Yanshuo Han, Zhaozhou Ren

**Affiliations:** ^1^School of Life and Pharmaceutical Sciences, Dalian University of Technology, Panjin 124001, China; ^2^Department of Orthopedic Surgery, The Third People's Hospital of Shenzhen, Shenzhen 518000, China; ^3^Department of Orthopaedic Surgery, Shengjing Hospital of China Medical University, Shenyang 110001, China

## Abstract

**Background:**

Inflammatory markers are associated with tumor genesis and progression, but their prognostic significance in osteosarcoma remains unclear. Therefore, we discussed the prognostic value of related inflammatory markers in osteosarcoma through a meta-analysis and systematic review. These inflammatory markers include C-reactive protein (CRP), neutrophil to lymphocyte ratio (NLR), lymphocyte to monocyte ratio (LMR), platelet to lymphocyte ratio (PLR), and Glasgow prognostic score (GPS).

**Methods:**

The Chinese National Knowledge Infrastructure (CNKI), Wanfang, Chinese Scientific Journals (VIP), PubMed, Embase, and Cochrane libraries were searched. The design of meta-analysis was made based on the PICOS (population, intervention/exposure, control, outcomes, and study design) principles, and STATA 15.1 was used to analyze the data. The Newcastle-Ottawa scale (NOS) was used to assess the quality of included studies. Hazard ratios (HRs) for overall survival (OS) and disease-specific survival (DPS) were extracted for the investigation of the prognostic value of inflammatory markers.

**Results:**

Twelve researches with 2162 osteosarcoma patients were included in total. The pooled results showed that elevated NLR, CRP, and GPS are all greatly related to shortening of OS among patients with osteosarcoma (HR = 1.68, *P* = 0.007, 95% CI: 1.15-2.45; HR = 1.96, *P* = 0.002, 95% CI: 1.28-3.00; HR = 2.54, *P* < 0.0001, 95% CI: 1.95-3.31, respectively), and CRP level is significantly associated with shortening of DPS among patients with osteosarcoma (HR = 2.76, 95% CI:2.01-3.80, *P* < 0.0001), additionally. However, the correlation between LMR or PLR and the prognosis of osteosarcoma is not statistically significant (HR = 0.60, 95% CI: 0.30-1.18, *P* = 0.138; HR = 1.13, 95% CI: 0.85-1.49, *P* = 0.405, respectively). The outcomes of subgroup analysis to NLR and CRP suggested that histology, ethnicity, metastasis, and sample size all have an impact on its prognosis of patients with osteosarcoma.

**Conclusion:**

Worsened prognosis may be related to high levels of NLR, CRP, and GPS before treatment rather than LMR or PLR, which can provide the basis for clinicians to judge the outcomes of prognosis. *Trial Registration*. PROSPERO (CRD42021249954), https://www.crd.york.ac.uk/prospero/display_record.php?RecordID=249954.

## 1. Introduction

As a greatly malignant bone tumor, osteosarcoma mainly influences adolescents and young adults, accounting for about 45% of all bone sarcomas [1]. The development of integrated chemotherapy in the 1970s increased the overall survival rates by about 50% [2]. Among them, the incidence of osteosarcoma in Europe is 7.3 per million person-years, while 12.2 per 1 million person-years in Asia [3]. In addition, the 5-year survival rate for osteosarcoma in Europe is 61% and 75% in Asia [4]. With the gradual development of clinical practice, the inaccuracy and inadequacy of traditional prognostic elements, such as the presence of tumor grade, metastasis, tumor location, and histological subtypes, have gradually been exposed [5]. Therefore, identifying more effective prognostic factors will be valuable for stratifying patients with different treatment options and improving survival.

In recent years, according to emerging evidence, systemic inflammatory response is an independent prognostic biomarker among different tumors. Moreover, according to increasing studies, there is a clear association between inflammatory markers and lower survival rates for some tumors such as neutrophil to lymphocyte ratio (NLR), Glasgow prognostic score (GPS), C-reactive protein (CRP), platelet to lymphocyte ratio (PLR), and lymphocyte to monocyte ratio (LMR) [6–9]. However, the predictive effect of these inflammatory indicators on the prognosis of osteosarcoma is unclear. The research of Liu et al. [10] and Xia et al. [11] believed that enhanced NLR is significantly related to the shortening of OS among patients with osteosarcoma, but the study of Huang et al. [12] suggested that NLR can be used as a protective factor for osteosarcoma. In addition, there is no significant relationship between CRP and the prognosis of osteosarcoma from the point of Li et al. [13] and Liu et al. [10]. Hence, the association between systemic inflammatory marker (e.g., NLR, CRP, LMR, GPS, and PLR) levels and the overall survival of patients with osteosarcoma was explored by a meta-analysis, aiming to assess these biomarkers as prognostic factors for overall survival and disease-specific survival.

## 2. Methods

The registration of systematic inspection at PROSPERO (http://www.crd.york.ac.uk/PROSPERO) as CRD42021249954 was made on basis of the associated items of the PRISMA statement [14].

### 2.1. Search Strategy

The English literatures of PubMed, Embase, and Cochrane libraries and the Chinese literature of CNKI, Wanfang, and VIP from their establishment to April 2021 will be comprehensively and systematically searched. PubMed, Cochrane Library, and Embase were searched through the subject words and keywords retrieval method using the following keywords: “Osteosarcoma” [MeSH], “C-reactive protein” [MeSH], “neutrophil to lymphocyte ratio” [MeSH], “Glasgow prognostic score” [MeSH], “lymphocyte to monocyte ratio” [MeSH], and “platelet to lymphocyte ratio” [MeSH] (Supplementary File 1). The manual retrieve of other associated articles was made from the reference lists or citations in the primary search or applying “Similar Articles” PubMed option. The CNKI, Wanfang, and VIP were searched using the general Chinese translation of the above search terms: C-reactive protein (CRP), neutrophil to lymphocyte ratio (NLR), lymphocyte to monocyte ratio (LMR), Glasgow prognostic score (GPS), and platelet to lymphocyte ratio (PLR).

### 2.2. Literature Inclusion and Exclusion Criteria

The eligibility criteria were mainly conducted in accordance with the PICOS (population, intervention/exposure, control, outcomes, and study design) principle limited to Chinese and English study.

The inclusion standards were shown below:

(a) *Population*. Patients with primary osteosarcoma who have survived radiation therapy, surgery, and chemotherapy

(b) *Exposure*. Risk factor (inflammatory marketer), including NLR, CRP, GPS, PLR, and LMR level

(c) *Comparators*. Normal levels of inflammatory markers in normal subjects

(d) *Outcomes*. Survival outcomes or clinicopathological characteristics of osteosarcoma cases, such as recurrence and metastasis

(e) *Study design*. Case-control study or cohort study

The following exclusion criteria were utilized: (a) papers which were meta-analysis, reviews, animal experiments, case reports, conference abstracts, non-English/Chinese literature, mechanism researches or other diseases/cancers, or lacking the full text; (b) duplicate publication or overlapped data which was offered in the prior article; (c) study provided insufficient information on survival outcomes about HR, or no data presented for CRP, NLR, GPS, PLR, and LMR level.

### 2.3. Literature Screening and Data Extraction

Independently, all eligibility surveys for inclusion in the study were conducted by two authors (X.S. and H.Z.), and any differences that arose during the screening process were discussed, negotiated, and resolved by the two authors together. In case of questions or controversies, the decision was made after discussing or consulting with a third person (Y.H.). For the data extraction, the author, publication year, study area, research type, number of cases, follow-up, and hazard ratios (Table 1) are for evaluating neutrophil-to-lymphocyte ratio (NLR), Glasgow prognostic score (GPS), C-reactive protein (CRP), platelet to lymphocyte ratio (PLR), and lymphocyte–monocyte ratio (LMR) of overall survival (OS) and disease-specific survival (DPS).

### 2.4. Literature Quality Assessment

Two researchers (X.S. and F.Y.) separately made literature quality evaluations applying the Newcastle-Ottawa Scale (NOS) for cohort study [15] in Table 2. There are 4 items (4 points) for “Research Subject Selection,” 1 item (2 points) for “Comparability between Groups,” and 3 items (3 points) for “Result Measurement” in NOS, with a full score of 9 points and ≥7 is regarded as high-quality literature, less than 7 is classified as low-quality literature.

### 2.5. Data Synthesis and Statistical Analysis

STATA version 15.1 statistical software (StataCorp LP, College Station, TX) was used to analyze the data. The association of associated inflammatory factors with OS and DPS was evaluated by using hazard ratios (HR) and 95% confidence intervals (CI). Heterogeneity was assessed by Cochran's *Q* statistic and *I*^2^. If the heterogeneity test is *P* ≥ 0.1 and *I*^2^ ≤ 50%, indicating the existence of homogeneity among the studies, and the combined analysis was made by the fixed-effect model; if *P* < 0.1, *I*^2^ > 50%, it indicates whether there is heterogeneity in the study. The source of heterogeneity was found by subgroup analysis based on race, histology, metastasis, and sample size. If the heterogeneity is still large, the random effects model was adopted or the combination of results was abandoned and descriptive analysis was adopted. Begg's test [16] and Egger's test [17] were utilized to estimate publication bias. Sensitivity analysis was utilized to estimate the robustness and reliability of the combined results influenced by a single included study.

## 3. Results

### 3.1. The Results of Literature Search

**Figure 1:**
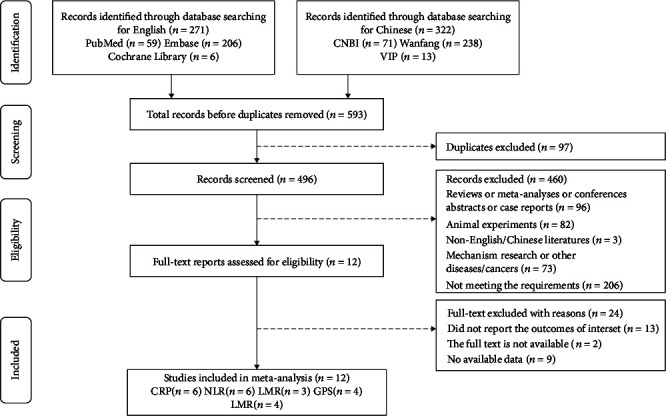
Flow chart of the study selection.

In this study, 593 studies were retrieved from the database in total. After eliminating duplicate studies, 97 were obtained. After browsing titles and abstracts, 36 researches were obtained. Finally, 12 articles meeting the requirements were brought into the meta-analysis (Figure 1), and there were 8 studies in China, 2 studies in British, 1 study in Denmark, and 1 study in Austria. The type of study included was a cohort study with a maximum follow-up time of 19 years (Table 1).

### 3.2. Systemic Inflammatory Markers and Overall Survival

**Figure 2:**
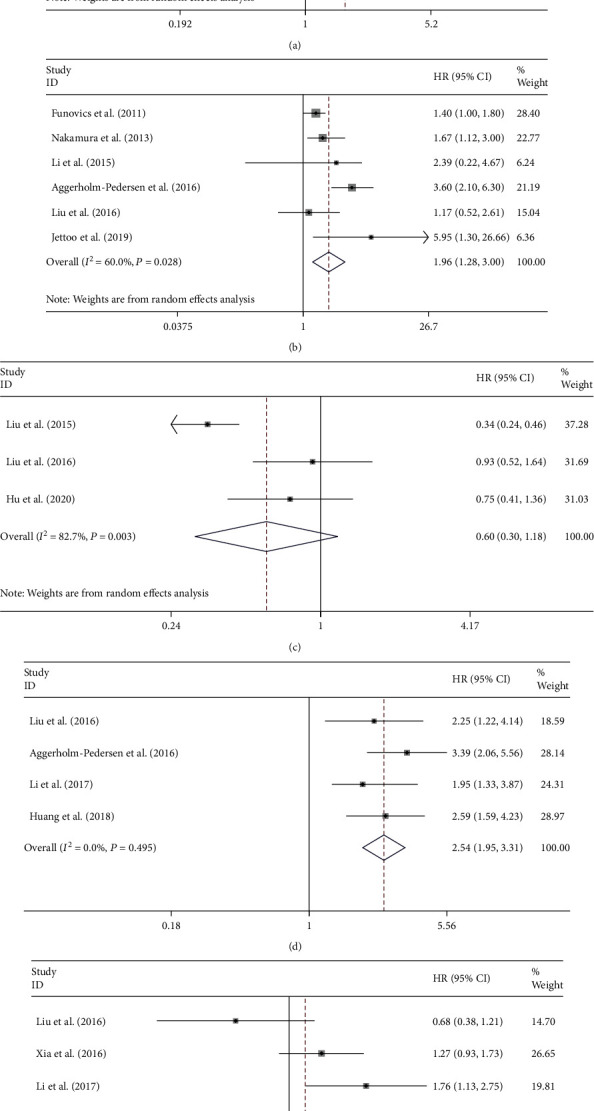
The association of NLR (a), CRP (b), LMR (c), GPS (d), and PLR (e) levels with the OS of patients with osteosarcoma.

6 studies reported an association between neutrophil-to-lymphocyte ratio (NLR) and overall survival among patients with osteosarcoma. With a meta-analysis conducted through a random-effects model, the pooled results show that elevated NLR is significantly associated with shortening of OS in patients with osteosarcoma (HR = 1.68, 95% CI: 1.15-2.45, *P* = 0.007; *I*^2^ = 84.7%, *P* < 0.0001; Figure 2(a)).

6 studies reported an association between C-reactive protein (CRP) and OS among patients with osteosarcoma. With a meta-analysis conducted through a random-effects model, the pooled results show that elevated CRP is significantly associated with shortening of OS in patients with osteosarcoma (HR = 1.96, 95% CI: 1.28-3.00, *P* = 0.002; *I*^2^ = 60.0%, *P* = 0.028; Figure 2(b)).

3 studies reported an association between lymphocyte–monocyte ratio (LMR) and OS among patients with osteosarcoma. With a meta-analysis conducted through a random effects model, the pooled results show that there is no significantly relationship between LMR and OS of patients with osteosarcoma (HR = 0.60, 95% CI: 0.30-1.18, *P* = 0.138; *I*^2^ = 82.7%, *P* = 0.003; Figure 2(c)).

4 studies reported an association between Glasgow prognostic score (GPS) and OS among patients with osteosarcoma. With a meta-analysis conducted through a fixed effects model, the pooled results show that GPS is significantly associated with shortening of OS among patients suffering from osteosarcoma (HR = 2.54, 95% CI: 1.95-3.31, *P* < 0.0001; *I*^2^ = 0.0%, *P* = 0.496; Figure 2(d)).

4 studies reported an association between platelet to lymphocyte ratio (PLR) and OS among patients with osteosarcoma. A meta-analysis was conducted through a random effects model, and the pooled results show that there is no significant relationship between PLR and OS of patients with osteosarcoma (HR = 1.13, 95% CI: 0.85-1.49, *P* = 0.405; *I*^2^ = 69.8%, *P* = 0.003; Figure 2(e)).

### 3.3. Systemic Inflammatory Markers and Disease-Specific Survival

**Figure 3:**
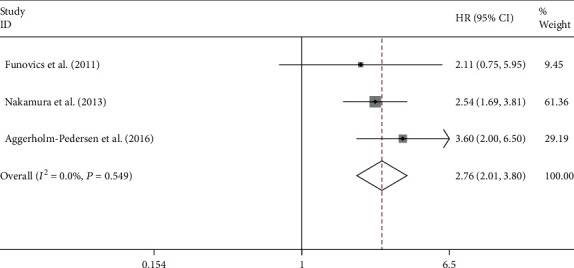
The association of CRP levels with the disease-specific survival of patients with osteosarcoma.

There were 3 studies that reported the relationship between C-reactive protein level and disease-specific survival (DPS). Additionally, the pooled results show that CRP is greatly related to the shortening of DPS among patients suffering from osteosarcoma (HR = 2.76, 95% CI: 2.01-3.80, *P* < 0.0001; *I*^2^ = 0.0%, *P* = 0.549; Figure 3).

### 3.4. Subgroup Analysis for Neutrophil-to-Lymphocyte Ratio

**Figure 4:**
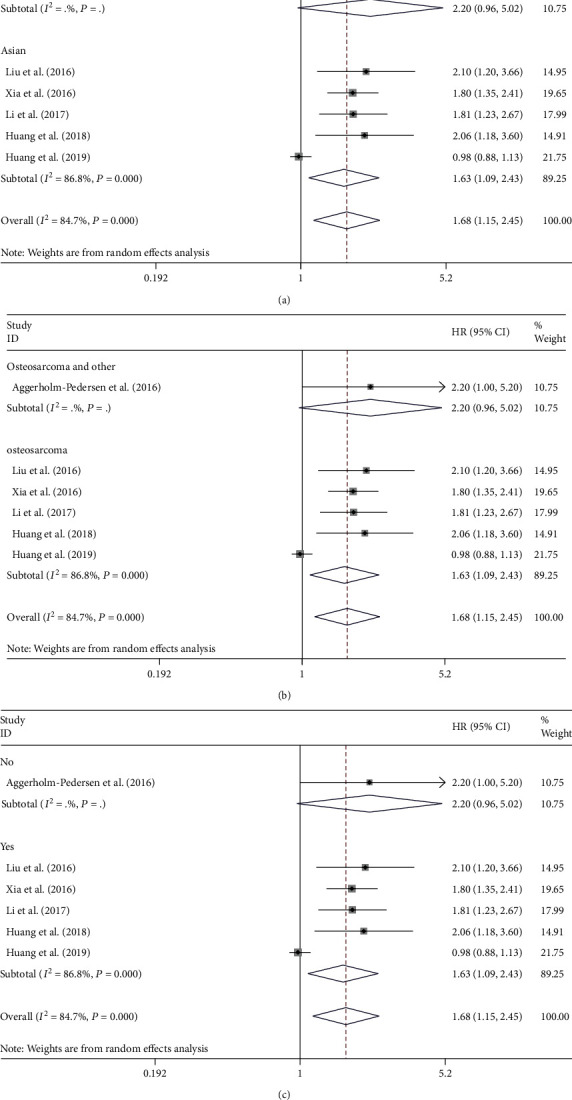
Subgroup analysis of the association of NLR levels with the OS of patients with osteosarcoma. The association of overall survival within European or Asian patients (a), osteosarcoma or other bone sarcomas (b), metastasis or nonmetastasis patients (c), and NLR levels with the OS of patients with osteosarcoma.

For the detection of the potential source of heterogeneity in analyzing the relation between NLR and OS, ethnicity, metastasis, histology, and sample size were applied to stratify the subgroup analysis. The pooled results show that the elevated NLR predicts poorer OS in Asian populations (HR = 1.63, 95% CI: 1.09-2.43, *P* = 0.017; Figure 4(a)), while the relationship between the level of NLR and OS was not significant in European populations (HR = 2.20, 95% CI: 0.96-5.02, *P* = 0.067; Figure 4(a)).

Subgroup analyses were also performed on histology and metastasis to further explain. Among patients suffering from osteosarcoma, growing NLR was related to shortened OS (HR = 1.63, 95% CI: 1.09-2.43, *P* = 0.017; Figure 4(b)). However, according to the pooled outcomes, there is no great relation between NLR and OS of patients suffering from osteosarcoma and other bone cancers (HR = 2.20, 95% CI: 0.96-5.02, *P* = 0.061; Figure 4(b)).

An enhanced level of NLR was related to reduced survival among patients with metastasis (HR = 1.63, 95% CI: 1.09-2.43, *P* = 0.017; Figure 4(c)), while the association between the level of NLR and OS was not evident in patients without metastasis (HR = 2.20, 95% CI: 0.96-5.02, *P* = 0.061; Figure 4(c)).

### 3.5. Subgroup Analysis for C-Reactive Protein

**Figure 5:**
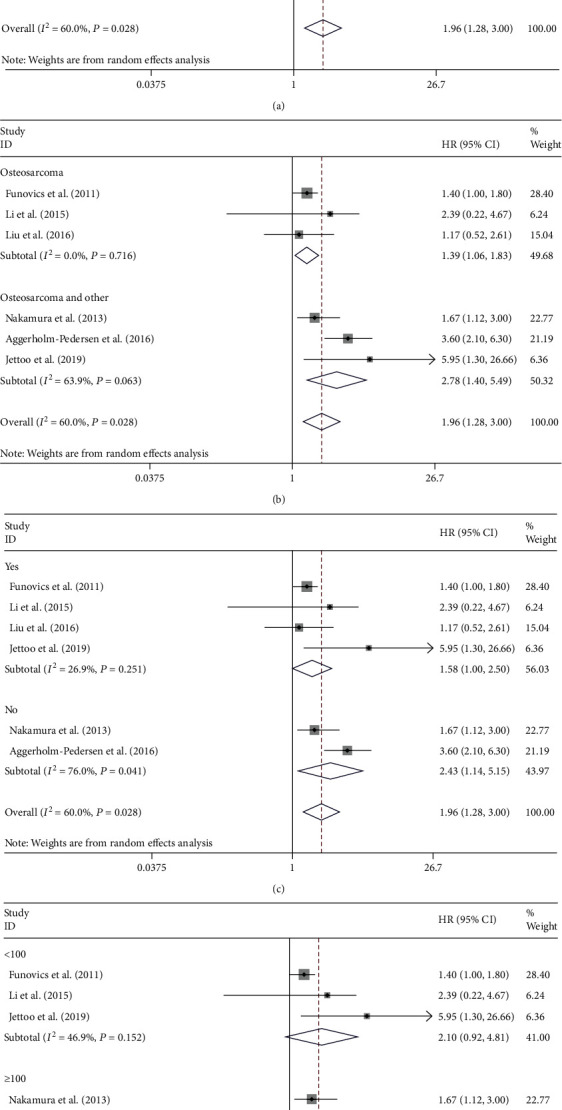
Subgroup analysis of the association of CRP levels with the OS of patients with osteosarcoma. The association of CRP level and overall survival within European or Asian patients (a), osteosarcoma or other bone sarcomas (b), metastasis or nonmetastasis patients (c), sample size (d) in patients with osteosarcoma.

Subgroup analysis for the detection of the potential source of heterogeneity in analyzing the association between CRP and OS was made by stratification by ethnicity, histology, metastasis, and sample size. The pooled results show that poorer OS in European populations (HR = 2.19, 95% CI: 1.28-3.74, *P* = 0.004; Figure 5(a)) can be predicted by the enhanced CRP level, while there was no great relationship between the level of CRP and OS in Asian populations (HR = 1.36, 95% CI: 0.67-2.78, *P* = 0.394; Figure 5(a)). Subgroup analyses on metastasis, histology, and sample size were performed for further explanation to further explain. Among patients suffering from osteosarcoma, increased CRP level was correlated with shortened OS (HR = 1.39, 95% CI: 1.06-1.83, *P* = 0.016; Figure 5(b)), and patients with bone sarcomas encountered the same situation (HR = 2.78; 95% CI: 1.40-5.49, *P* = 0.003; Figure 5(b)). An enhanced level of CRP was related to reduced survival among patients with or without metastasis (Figure 5(c)). Additionally, the pooled outcomes displayed that in studies with a sample size of greater than or equal to 100 patients, elevated CRP predicted poor OS (HR = 1.99, 95% CI: 1.06-3.74, *P* = 0.032; Figure 5(d)). However, in a sample size less than 100, relationship between CRP and OS was not significant (HR = 2.10, 95% CI: 0.92-4.81, *P* = 0.080; Figure 5(d)).

### 3.6. Sensitivity Analysis

Sensitivity analysis eliminated every included research successively and performed a summary discussion on the remaining researches to evaluate whether a single included research excessively influenced on the overall outcomes of the meta-analysis. The outcomes of the sensitivity analysis are shown in Supplementary file 2, indicating that no research exerted an excessive impact on the outcomes of the meta-analysis, and that the outcomes of the remaining researches are stable and credible.

### 3.7. Publication Bias

**Figure 6:**
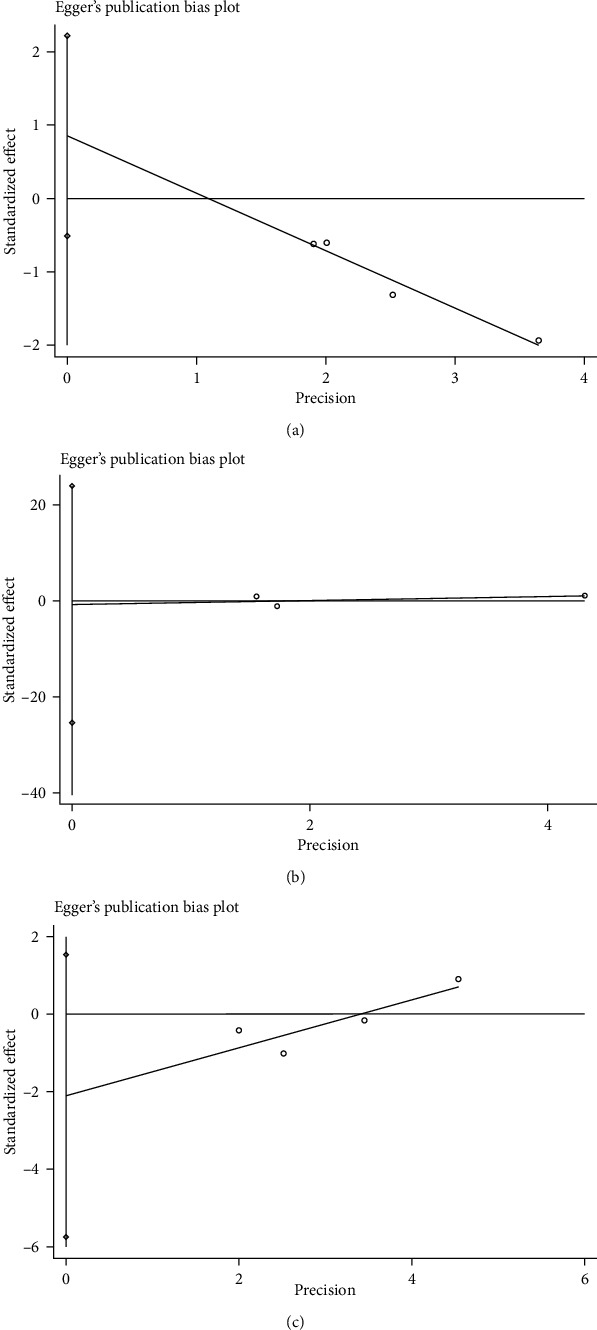
Egger's test of the association of NLR (a), CRP (b), and GPS (c) levels with the OS of patients with osteosarcoma.

The Begg's funnel plot of this study is shown in Supplementary file 3. It could be seen that the funnel plot was basically symmetrical, and the *P* value of Egger's test for NLR was 0.115 (Figure 6), for CRP was 0.762, for GPS was 0.130, indicating that no obvious publication bias in this study.

## 4. Discussion

This meta-analysis pooled 12 researches, including 2,162 patients, to examine the relation between C-reactive protein (CRP), neutrophil to lymphocyte ratio (NLR), lymphocyte to monocyte ratio (LMR), Glasgow prognostic score (GPS), and platelet to lymphocyte ratio (PLR) levels with the OS of patients suffering from osteosarcoma, aiming to assess these biomarkers as prognostic elements for overall survival and disease-specific survival.

Inflammation is essential for human tumors, malignant transformation, and antitumor immunity [26]. It is increasingly recognized that systemic inflammation exerts a vital effect on the occurrence and growth of cancer [27, 28]. Inflammatory factors can directly provide free radicals to attack normal DNA mechanisms and cause cancer or indirectly damage DNA and regulate gene expression by affecting the epigenetic characteristics of cells [29]. Neoplastic cells often excessively express proinflammatory mediators such as proteases, cytokines, and chemokines [30]. Various types of oncogenes are activated through mutation, chromosomal rearrangement, or amplification. Transformed cells undergoing this process produce inflammatory mediators that activate the expression of transcription factors. Then, activated transcription factors further coordinate the production of inflammatory mediators and ultimately form cancer-related microenvironments [27]. It is not surprising to detect increased levels of CRP, NLR, or GPS in cancer because of the importance of the inflammation in the development of cancer.

It is known that NLR values increase in acute pancreatitis [31], cardiac events [32], and atherosclerosis [33]. As a marker of systemic inflammation, NLR can also be considered as a potential prognostic factor for different tumors. Pretreatment NLR was utilized as a prognostic indicator of long-term mortality in patients with breast cancer by Azab et al. [34]. According to Deng et al., preoperative NLR is a separate prognostic factor specific to cancer survival among patients undergoing gastric cancer surgery [9]. However, the relation between NLR and the prognosis of patients suffering from osteosarcoma remains controversial. The pooled outcomes showed that enhanced NLR is greatly related to the shortening of OS in patients with osteosarcoma, showing that great serum levels of NLR before treatment may be a negative prognostic element for patients suffering from bone cancers.

It has been also shown that increased levels of systemic inflammation are related to lower survival rates in patients with solid tumors [35, 36]. CRP is a nonspecific but sensitive marker of systemic inflammation synthesized by liver cells replying microbial invasion or tissue damage [37]. It is well known that during inflammation, acute infection, and tissue damage, CRP levels will rise rapidly. In addition, enhanced CRP levels are also regarded as a significant risk element for atherosclerosis [38], stroke [39–41], and myocardial infarction [42]. Importantly, it has been confirmed that the preoperative level of serum CRP is related to the prognosis of hepatocellular carcinoma [43] and pancreatic cancer [44]. Our pooled results also found that elevated levels of CRP are greatly related to the shortening of OS in patients with osteosarcoma (HR = 1.96, 95% CI: 1.28-3.00) which conforms to the outcomes of most researches [13, 18, 19, 21]. Additionally, pooled result showed that CRP is significantly associated with shortening of DPS in patients with osteosarcoma (HR = 2.76, 95% CI: 2.01-3.80). These all suggested that CRP is a risk factor for the prognosis of osteosarcoma. To improve the prognosis of the patients with elevated CRP, NLR, and GPS, it is urgently needed a management protocol for systemic inflammatory response via the tumor-host interaction during the postoperative course is urgently needed to improve their prognosis [45].

Simultaneously, no great association between the level of NLR and OS in Europe patients was found in the stratified analysis (HR = 2.20, 95% CI: 0.96-5.02) and no significant association between CRP levels and OS in Asia patients (HR = 1.36, 95% CI: 0.67-2.78). This may be due to the differences in the susceptibility genes, treatment options, and CRP measurement methods of bone tumors in Asia and Europe. Subgroup analyses on metastasis, histology, and sample size were made for the explanation of heterogeneity. In the analysis of NLR, the pooled results showed that there is no significant relationship between NLR and OS of patients suffering from osteosarcoma and other bone cancers (HR = 2.20, 95% CI: 0.96-5.02). The similar result also appeared in nonmetastasis osteosarcoma, indicating that histology and metastasis may be the cause of high heterogeneity. In the analysis of CRP, enhanced CRP was related to shortened OS in patients with osteosarcoma (HR = 1.39, 95% CI: 1.06-1.83), and patients with other bone cancers encountered the same situation. Similarly, the pooled results showed that an enhanced level of CRP was related to reduced OS in patients with osteosarcoma regardless of metastasis. The results suggested that this high heterogeneity may be independent of histology and metastasis.

The definition of GPS was carried out on the basis of the presence of hypoalbuminemia (<35 g/L) and enhanced CRP (>10 mg/L): if both were abnormal, the score was 2; if either was abnormal, the score was 1; if there were no exceptions, the score was 0 [46, 47]. According to increasing researches, the hidden predictive value of GPS was demonstrated among osteosarcoma patients. One study speculates that GPS shows inflammation status and nutritional status of cancer patients as a better predictor of prognosing cancer than CRP [10]. Hence, this systematic examination and meta-analysis shall be made to draw more reliable conclusions on the effect of GPS on osteosarcoma. In this meta-analysis, measuring GPS was an effective way to predict prognosis among patients suffering from osteosarcoma. Additionally, according to the pooled results, GPS is significantly associated with shortening of OS in patients with osteosarcoma (HR = 2.54, 95% CI: 1.95-3.31), demonstrating that high level of GPS before treatment may also be a negative prognostic element for patients with osteosarcoma.

The prognostic value of PLR and LMR for other tumors shows different conclusions from this article. In the latest study, PLR is thought to be inversely associated with the prognosis of breast cancer [48], and LMR is considered as a risk factor for gastric cancer [49]. The pooled results show that there is no significant relationship between PLR or LMR and OS of patients with osteosarcoma. This anomaly of LMR may be related to the insufficient number of included studies; in addition to this, there remains a study for PLR showing that the predictive value of high PLR in terms of overall survival is greater in cancer patients with comorbidities, especially those with metabolic syndrome [50] which may not be consistent with patients with osteosarcoma. In addition, due to the error of measurement results and the influence of other unrelated confounding factors, some research results may be ignored and reported, resulting in the trend of the prognostic value of these two inflammatory markers for osteosarcoma is not obvious. However, specific conclusions need to be supported by further research results.

Different types of treatment and osteosarcoma may have different overall survival times, which may contribute to high heterogeneity. Due to the differences between individuals and groups, patients of different races, regions, and ages may have different degrees of disease or disease tendency, resulting in different treatment methods. This leads to the blending of various factors, which we cannot distinguish in detail for the time being. We believe that there will be more scientific statistical methods and more rigorous experimental design to solve these problems in the future.

In addition to the above problems, this meta-analysis does have several limitations. First, most of the included studies were retrospectively designed, which increased the risk of bias due to inadequate random blinding. Second, even though subgroup analyses were performed, there was an obvious heterogeneity in this meta-analysis, but at present, we have not yet found a clear cause for the heterogeneity. Third, the overall results may be overestimated because of negative data from unpublished studies. Fourth, restricted by insufficient number of literature and the original data, the reliability of the results may be shortened and we cannot draw receiver operating characteristic curves (ROC curves) to study the prognostic value of each marker, further. We can only expect more studies to be carried out so that we can update this meta-analysis. Finally, sensitivity analysis and funnel plots showed potential publication bias in some researches. After ignoring these researches, the distribution of OS in the remaining studies was more symmetrical. This bias may be due to differences in baseline characteristics and study regimen-related protocols among patients. Moreover, the differences in detection methods and data storage may have resulted in heterogeneity. Although the random effects model reduced the effect of heterogeneity, the heterogeneity between studies was not abolished. In view of the above limitations, it is recommended to prospectively recruit subjects in future studies. At the same time, researchers may consider combining multiple inflammatory markers to explore their common prognostic value and make the results more sensitive.

## 5. Conclusion

For patients with osteosarcoma, meta-analysis performed in this paper demonstrated that high great contents of NLR, CRP, and GPS before treatment may be a negative prognostic element, and ethnicity, histology, and metastasis all have an impact on its prognosis of patients with osteosarcoma; however, PLR and LMR might have nothing to do with it. In conclusion, the measurement of these inflammatory markers' levels can provide the basis for clinicians to judge the outcome of prognosis.

## Figures and Tables

**Table 1:** Baseline characteristics and quality assessment of the included studies.

Study	Year	Location	Sample size	Type	Metastasis	Follow-up^∗^	Gender (m/f)	Treatment	Outcomes	Inflammatory markers
Funovics et al. [18]	2011	Austria	79	Osteosarcoma	30/49	33 (1-126) months	42/37	Chemotherapy; surgery	DPS/OS	CRP
Nakamura et al. [19]	2013	British	318	Osteosarcoma; Ewing's sarcoma; chondrosarcoma	NO	40 (1-109) months	176/142	Radiotherapy; surgery; chemotherapy	OS	CRP
Liu et al. [20]	2015	China	327	Osteosarcoma	130/197	24 (3-60) months	235/92	Surgery; chemotherapy	OS	LMR
Liu et al. [10]	2016	China	162	Osteosarcoma	78/162	NA	96/66	Chemotherapy; surgery	OS	CRP, GPS, LMR, NLR, PLR
Aggerholm-Pedersen et al. [21]	2016	Denmark	172	Chondrosarcoma; Ewing's sarcoma; osteosarcoma	NO	8.8 (4.3-19) years	98/74	Radiotherapy; surgery; chemotherapy	DPS/OS	CRP, GPS, NLR
Xia et al. [11]	2016	China	359	Osteosarcoma	132/227	40 (3-60) months	258/101	Surgery; chemotherapy	OS	NLR, PLR,
Li et al. [22]	2017	China	215	Osteosarcoma	30/49	NA	122/94	Surgery; chemotherapy	OS	GPS, NLR, PLR
Li et al. [13]	2015	China	85	Osteosarcoma	37/85	NA	43/42	Chemotherapy	OS	CRP
Jettoo et al. [23]	2019	British	79	Osteosarcoma; chondrosarcoma; Ewing's sarcoma	14/63	45 (1-172) months	44/35	Surgery; chemotherapy	DPS/OS	CRP
Huang et al. [24]	2018	China	103	Osteosarcoma	13/90	43 months	63/40	Surgery; chemotherapy	OS	GPS, NLR
Huang et al. [12]	2019	China	126	Osteosarcoma	7/119	44 (7-81) months	78/48	Surgery; chemotherapy	OS	NLR, PLR
Hu et al. [25]	2020	China	137	Osteosarcoma	61/76	55 (4-112) months	80/57	Surgery; chemotherapy	OS	LMR

Note: “∗” median (minimum-maximum) follow-up time; NA: data were not provided in the publication.

**Table 2:** Newcastle-Ottawa scale (NOS) of cohort studies.

Study	Year	Selection				Comparability	Outcome			Score
		Representativeness of exposed cohort	Selection of the nonexposed cohort	Ascertainment of exposure	Outcome of interest was not present	Comparability of exposure and nonexposure	Ascertainment of outcome	Follow-up enough for outcome	Adequacy of follow-up	
Funovics et al. [18]	2011	★		★	★	★	★	★	★	7
Hu et al. [25]	2020	★	★	★	★	★	★	★	★	8
Huang et al. [12]	2019	★	★	★	★	★	★	★	★	8
Jettoo et al. [23]	2019	★	★	★	★	★	★	★	★	8
Aggerholm-Pedersen et al. [21]	2016	★		★	★	★	★	★	★	7
Li et al. [13]	2015	★	★	★	★	★		★		6
Li et al. [22]	2017	★	★	★	★	★	★	★	★	8
Liu et al. [10]	2016	★	★	★	★	★	★	★	★	8
Liu et al. [20]	2015	★		★	★	★	★	★	★	7
Nakamura et al. [19]	2013	★		★	★	★	★	★	★	7
Xia et al. [11]	2016	★	★	★	★	★	★	★	★	8
Huang et al. [24]	2018	★	★	★	★	★		★	★	7

## Data Availability

The datasets are available from the corresponding author on reasonable request.

## Additional Points

*Code Availability*. Microsoft Excel 2010; Microsoft, Redmond, WA, USA. STATA meta. Ado module (version 15.1; College Station, TX, USA).

## Supplementary Materials

Supplementary Materials Supplementary File 1: search strategy for Medline database. Supplementary File 2: sensitivity analysis of the association of NLR, CRP, LMR, GRS, and PLR levels with the OS of patients with osteosarcoma. Supplementary File 3: Begg's funnel plot of the association of NLR, CRP, and GPS levels with the OS of patients with osteosarcoma.
