# Supplementary material for: Systemic Inflammatory Markers for Predicting Overall Survival in Patients with Osteosarcoma: A Systematic Review and Meta-Analysis
Source: Mediators Inflamm. 2021 Oct 21;2021:3456629. doi: 10.1155/2021/3456629 (PMC8553478; doi:10.1155/2021/3456629)
Supplement: Supplementary Materials — Supplementary File 1: search strategy for Medline database. Supplementary File 2: sensitivity analysis of the association of NLR, CRP, LMR, GRS, and PLR levels with the OS of patients with osteosarcoma. Supplementary File 3: Begg's funnel plot of the association of NLR, CRP, and GPS levels with the OS of patients with osteosarcoma. [file 3456629.f1.zip › 4.Supplementary File 1.docx]

**Supplementary file 1.**

| Search number | Search Details | Results |
| --- | --- | --- |
| 1 | "Osteosarcoma"[MeSH Terms] | 29,110 |
| 2 | "osteosarcoma"[MeSH Terms] OR "osteosarcoma"[All Fields] OR "osteosarcomas"[All Fields] | 38,405 |
| 3 | "osteosarcoma"[MeSH Terms] OR "osteosarcoma"[All Fields] OR "osteosarcomas"[All Fields] | 38,405 |
| 4 | "osteosarcoma"[MeSH Terms] OR "osteosarcoma"[All Fields] OR ("osteosarcoma"[All Fields] AND "tumor"[All Fields]) OR "osteosarcoma tumor"[All Fields] | 37,894 |
| 5 | "osteosarcoma"[MeSH Terms] OR "osteosarcoma"[All Fields] OR ("osteosarcoma"[All Fields] AND "tumors"[All Fields]) OR "osteosarcoma tumors"[All Fields] | 37,894 |
| 6 | "osteosarcoma"[MeSH Terms] OR "osteosarcoma"[All Fields] OR ("tumor"[All Fields] AND "osteosarcoma"[All Fields]) OR "tumor osteosarcoma"[All Fields] | 37,894 |
| 7 | "osteosarcoma"[MeSH Terms] OR "osteosarcoma"[All Fields] OR ("tumors"[All Fields] AND "osteosarcoma"[All Fields]) OR "tumors osteosarcoma"[All Fields] | 37,894 |
| 8 | "osteosarcoma"[MeSH Terms] OR "osteosarcoma"[All Fields] OR ("sarcoma"[All Fields] AND "osteogenic"[All Fields]) OR "sarcoma osteogenic"[All Fields] | 38,450 |
| 9 | "osteosarcoma"[MeSH Terms] OR "osteosarcoma"[All Fields] OR ("osteogenic"[All Fields] AND "sarcomas"[All Fields]) OR "osteogenic sarcomas"[All Fields] | 38,028 |
| 10 | "osteosarcoma"[MeSH Terms] OR "osteosarcoma"[All Fields] OR ("sarcomas"[All Fields] AND "osteogenic"[All Fields]) OR "sarcomas osteogenic"[All Fields] | 38,028 |
| 11 | "osteosarcoma"[MeSH Terms] OR "osteosarcoma"[All Fields] OR ("osteogenic"[All Fields] AND "sarcoma"[All Fields]) OR "osteogenic sarcoma"[All Fields] | 38,450 |
| 12 | "Osteosarcoma"[MeSH Terms] OR ("Osteosarcoma"[MeSH Terms] OR "Osteosarcoma"[All Fields] OR "osteosarcomas"[All Fields]) OR ("Osteosarcoma"[MeSH Terms] OR "Osteosarcoma"[All Fields] OR "osteosarcomas"[All Fields]) OR ("Osteosarcoma"[MeSH Terms] OR "Osteosarcoma"[All Fields] OR ("Osteosarcoma"[All Fields] AND "tumor"[All Fields]) OR "osteosarcoma tumor"[All Fields]) OR ("Osteosarcoma"[MeSH Terms] OR "Osteosarcoma"[All Fields] OR ("Osteosarcoma"[All Fields] AND "tumors"[All Fields]) OR "osteosarcoma tumors"[All Fields]) OR ("Osteosarcoma"[MeSH Terms] OR "Osteosarcoma"[All Fields] OR ("tumor"[All Fields] AND "Osteosarcoma"[All Fields]) OR "tumor osteosarcoma"[All Fields]) OR ("Osteosarcoma"[MeSH Terms] OR "Osteosarcoma"[All Fields] OR ("tumors"[All Fields] AND "Osteosarcoma"[All Fields]) OR "tumors osteosarcoma"[All Fields]) OR ("Osteosarcoma"[MeSH Terms] OR "Osteosarcoma"[All Fields] OR ("sarcoma"[All Fields] AND "osteogenic"[All Fields]) OR "sarcoma osteogenic"[All Fields]) OR ("Osteosarcoma"[MeSH Terms] OR "Osteosarcoma"[All Fields] OR ("osteogenic"[All Fields] AND "sarcomas"[All Fields]) OR "osteogenic sarcomas"[All Fields]) OR ("Osteosarcoma"[MeSH Terms] OR "Osteosarcoma"[All Fields] OR ("sarcomas"[All Fields] AND "osteogenic"[All Fields]) OR "sarcomas osteogenic"[All Fields]) OR ("Osteosarcoma"[MeSH Terms] OR "Osteosarcoma"[All Fields] OR ("osteogenic"[All Fields] AND "sarcoma"[All Fields]) OR "osteogenic sarcoma"[All Fields]) | 38,997 |
| 13 | "C-Reactive Protein"[MeSH Terms] | 47,669 |
| 14 | "c reactive protein"[MeSH Terms] OR ("c reactive"[All Fields] AND "protein"[All Fields]) OR "c reactive protein"[All Fields] OR "c reactive protein"[All Fields] | 85,629 |
| 15 | "c reactive protein"[MeSH Terms] OR ("c reactive"[All Fields] AND "protein"[All Fields]) OR "c reactive protein"[All Fields] OR "c reactive protein"[All Fields] | 85,629 |
| 16 | "c reactive protein"[MeSH Terms] OR ("c reactive"[All Fields] AND "protein"[All Fields]) OR "c reactive protein"[All Fields] OR "hscrp"[All Fields] | 87,454 |
| 17 | "c reactive protein"[MeSH Terms] OR ("c reactive"[All Fields] AND "protein"[All Fields]) OR "c reactive protein"[All Fields] OR "high sensitivity c reactive protein"[All Fields] | 85,629 |
| 18 | "c reactive protein"[MeSH Terms] OR ("c reactive"[All Fields] AND "protein"[All Fields]) OR "c reactive protein"[All Fields] OR "high sensitivity c reactive protein"[All Fields] | 85,629 |
| 19 | "c reactive protein"[MeSH Terms] OR ("c reactive"[All Fields] AND "protein"[All Fields]) OR "c reactive protein"[All Fields] OR ("hs"[All Fields] AND "crp"[All Fields]) OR "hs crp"[All Fields] | 86,735 |
| 20 | "curr res psychol"[Journal] OR "crp"[All Fields] | 53,058 |
| 21 | "C-Reactive Protein"[MeSH Terms] OR ("C-Reactive Protein"[MeSH Terms] OR ("c reactive"[All Fields] AND "protein"[All Fields]) OR "C-Reactive Protein"[All Fields] OR "C-Reactive Protein"[All Fields]) OR ("C-Reactive Protein"[MeSH Terms] OR ("c reactive"[All Fields] AND "protein"[All Fields]) OR "C-Reactive Protein"[All Fields] OR "C-Reactive Protein"[All Fields]) OR ("C-Reactive Protein"[MeSH Terms] OR ("c reactive"[All Fields] AND "protein"[All Fields]) OR "C-Reactive Protein"[All Fields] OR "hscrp"[All Fields]) OR ("C-Reactive Protein"[MeSH Terms] OR ("c reactive"[All Fields] AND "protein"[All Fields]) OR "C-Reactive Protein"[All Fields] OR "high sensitivity c reactive protein"[All Fields]) OR ("C-Reactive Protein"[MeSH Terms] OR ("c reactive"[All Fields] AND "protein"[All Fields]) OR "C-Reactive Protein"[All Fields] OR "high sensitivity c reactive protein"[All Fields]) OR ("C-Reactive Protein"[MeSH Terms] OR ("c reactive"[All Fields] AND "protein"[All Fields]) OR "C-Reactive Protein"[All Fields] OR ("hs"[All Fields] AND "crp"[All Fields]) OR "hs crp"[All Fields]) OR ("curr res psychol"[Journal] OR "crp"[All Fields]) | 99,949 |
| 22 | ("neutrophil s"[All Fields] OR "neutrophiles"[All Fields] OR "neutrophilic"[All Fields] OR "neutrophillic"[All Fields] OR "neutrophils"[MeSH Terms] OR "neutrophils"[All Fields] OR "neutrophil"[All Fields] OR "neutrophile"[All Fields]) AND ("lymphocytes"[MeSH Terms] OR "lymphocytes"[All Fields] OR "lymphocyte count"[MeSH Terms] OR ("lymphocyte"[All Fields] AND "count"[All Fields]) OR "lymphocyte count"[All Fields] OR "lymphocyte"[All Fields] OR "lymphocytic"[All Fields] OR "lymphocyts"[All Fields]) AND ("ratio"[All Fields] OR "ratio s"[All Fields] OR "ratioes"[All Fields] OR "ratios"[All Fields]) | 9,386 |
| 23 | "NLR"[All Fields] | 13,321 |
| 25 | "PLR"[All Fields] | 3,778 |
| 26 | ("blood platelets"[MeSH Terms] OR ("blood"[All Fields] AND "platelets"[All Fields]) OR "blood platelets"[All Fields] OR "platelet"[All Fields] OR "platelets"[All Fields] OR "platelet s"[All Fields] OR "plateletes"[All Fields]) AND ("lymphocytes"[MeSH Terms] OR "lymphocytes"[All Fields] OR "lymphocyte count"[MeSH Terms] OR ("lymphocyte"[All Fields] AND "count"[All Fields]) OR "lymphocyte count"[All Fields] OR "lymphocyte"[All Fields] OR "lymphocytic"[All Fields] OR "lymphocyts"[All Fields]) AND ("ratio"[All Fields] OR "ratio s"[All Fields] OR "ratioes"[All Fields] OR "ratios"[All Fields]) | 4,009 |
| 27 | "GPS"[All Fields] | 26,818 |
| 28 | ("glasgow"[All Fields] OR "glasgow s"[All Fields]) AND ("prognostic"[All Fields] OR "prognostical"[All Fields] OR "prognostically"[All Fields] OR "prognosticate"[All Fields] OR "prognosticated"[All Fields] OR "prognosticates"[All Fields] OR "prognosticating"[All Fields] OR "prognostication"[All Fields] OR "prognostications"[All Fields] OR "prognosticator"[All Fields] OR "prognosticators"[All Fields] OR "prognostics"[All Fields]) AND ("score"[All Fields] OR "score s"[All Fields] OR "scored"[All Fields] OR "scores"[All Fields] OR "scoring"[All Fields] OR "scorings"[All Fields]) | 2,819 |
| 29 | "LMR"[All Fields] | 1,565 |
| 30 | ("lymphocytes"[MeSH Terms] OR "lymphocytes"[All Fields] OR "lymphocyte count"[MeSH Terms] OR ("lymphocyte"[All Fields] AND "count"[All Fields]) OR "lymphocyte count"[All Fields] OR "lymphocyte"[All Fields] OR "lymphocytic"[All Fields] OR "lymphocyts"[All Fields]) AND ("monocyte s"[All Fields] OR "monocytes"[MeSH Terms] OR "monocytes"[All Fields] OR "monocyte"[All Fields] OR "monocytic"[All Fields]) AND ("ratio"[All Fields] OR "ratio s"[All Fields] OR "ratioes"[All Fields] OR "ratios"[All Fields]) | 3,645 |
| 31 | (("neutrophil s"[All Fields] OR "neutrophiles"[All Fields] OR "neutrophilic"[All Fields] OR "neutrophillic"[All Fields] OR "neutrophils"[MeSH Terms] OR "neutrophils"[All Fields] OR "neutrophil"[All Fields] OR "neutrophile"[All Fields]) AND ("lymphocytes"[MeSH Terms] OR "lymphocytes"[All Fields] OR "lymphocyte count"[MeSH Terms] OR ("lymphocyte"[All Fields] AND "count"[All Fields]) OR "lymphocyte count"[All Fields] OR "lymphocyte"[All Fields] OR "lymphocytic"[All Fields] OR "lymphocyts"[All Fields]) AND ("ratio"[All Fields] OR "ratio s"[All Fields] OR "ratioes"[All Fields] OR "ratios"[All Fields])) OR "NLR"[All Fields] OR "PLR"[All Fields] OR (("blood platelets"[MeSH Terms] OR ("blood"[All Fields] AND "platelets"[All Fields]) OR "blood platelets"[All Fields] OR "platelet"[All Fields] OR "platelets"[All Fields] OR "platelet s"[All Fields] OR "plateletes"[All Fields]) AND ("lymphocytes"[MeSH Terms] OR "lymphocytes"[All Fields] OR "lymphocyte count"[MeSH Terms] OR ("lymphocyte"[All Fields] AND "count"[All Fields]) OR "lymphocyte count"[All Fields] OR "lymphocyte"[All Fields] OR "lymphocytic"[All Fields] OR "lymphocyts"[All Fields]) AND ("ratio"[All Fields] OR "ratio s"[All Fields] OR "ratioes"[All Fields] OR "ratios"[All Fields])) OR "GPS"[All Fields] OR (("glasgow"[All Fields] OR "glasgow s"[All Fields]) AND ("prognostic"[All Fields] OR "prognostical"[All Fields] OR "prognostically"[All Fields] OR "prognosticate"[All Fields] OR "prognosticated"[All Fields] OR "prognosticates"[All Fields] OR "prognosticating"[All Fields] OR "prognostication"[All Fields] OR "prognostications"[All Fields] OR "prognosticator"[All Fields] OR "prognosticators"[All Fields] OR "prognostics"[All Fields]) AND ("score"[All Fields] OR "score s"[All Fields] OR "scored"[All Fields] OR "scores"[All Fields] OR "scoring"[All Fields] OR "scorings"[All Fields])) OR "LMR"[All Fields] OR (("lymphocytes"[MeSH Terms] OR "lymphocytes"[All Fields] OR "lymphocyte count"[MeSH Terms] OR ("lymphocyte"[All Fields] AND "count"[All Fields]) OR "lymphocyte count"[All Fields] OR "lymphocyte"[All Fields] OR "lymphocytic"[All Fields] OR "lymphocyts"[All Fields]) AND ("monocyte s"[All Fields] OR "monocytes"[MeSH Terms] OR "monocytes"[All Fields] OR "monocyte"[All Fields] OR "monocytic"[All Fields]) AND ("ratio"[All Fields] OR "ratio s"[All Fields] OR "ratioes"[All Fields] OR "ratios"[All Fields])) | 51,552 |
| 32 | (("neutrophil s"[All Fields] OR "neutrophiles"[All Fields] OR "neutrophilic"[All Fields] OR "neutrophillic"[All Fields] OR "neutrophils"[MeSH Terms] OR "neutrophils"[All Fields] OR "neutrophil"[All Fields] OR "neutrophile"[All Fields]) AND ("lymphocytes"[MeSH Terms] OR "lymphocytes"[All Fields] OR "lymphocyte count"[MeSH Terms] OR ("lymphocyte"[All Fields] AND "count"[All Fields]) OR "lymphocyte count"[All Fields] OR "lymphocyte"[All Fields] OR "lymphocytic"[All Fields] OR "lymphocyts"[All Fields]) AND ("ratio"[All Fields] OR "ratio s"[All Fields] OR "ratioes"[All Fields] OR "ratios"[All Fields])) OR "NLR"[All Fields] OR "PLR"[All Fields] OR (("blood platelets"[MeSH Terms] OR ("blood"[All Fields] AND "platelets"[All Fields]) OR "blood platelets"[All Fields] OR "platelet"[All Fields] OR "platelets"[All Fields] OR "platelet s"[All Fields] OR "plateletes"[All Fields]) AND ("lymphocytes"[MeSH Terms] OR "lymphocytes"[All Fields] OR "lymphocyte count"[MeSH Terms] OR ("lymphocyte"[All Fields] AND "count"[All Fields]) OR "lymphocyte count"[All Fields] OR "lymphocyte"[All Fields] OR "lymphocytic"[All Fields] OR "lymphocyts"[All Fields]) AND ("ratio"[All Fields] OR "ratio s"[All Fields] OR "ratioes"[All Fields] OR "ratios"[All Fields])) OR "GPS"[All Fields] OR (("glasgow"[All Fields] OR "glasgow s"[All Fields]) AND ("prognostic"[All Fields] OR "prognostical"[All Fields] OR "prognostically"[All Fields] OR "prognosticate"[All Fields] OR "prognosticated"[All Fields] OR "prognosticates"[All Fields] OR "prognosticating"[All Fields] OR "prognostication"[All Fields] OR "prognostications"[All Fields] OR "prognosticator"[All Fields] OR "prognosticators"[All Fields] OR "prognostics"[All Fields]) AND ("score"[All Fields] OR "score s"[All Fields] OR "scored"[All Fields] OR "scores"[All Fields] OR "scoring"[All Fields] OR "scorings"[All Fields])) OR "LMR"[All Fields] OR (("lymphocytes"[MeSH Terms] OR "lymphocytes"[All Fields] OR "lymphocyte count"[MeSH Terms] OR ("lymphocyte"[All Fields] AND "count"[All Fields]) OR "lymphocyte count"[All Fields] OR "lymphocyte"[All Fields] OR "lymphocytic"[All Fields] OR "lymphocyts"[All Fields]) AND ("monocyte s"[All Fields] OR "monocytes"[MeSH Terms] OR "monocytes"[All Fields] OR "monocyte"[All Fields] OR "monocytic"[All Fields]) AND ("ratio"[All Fields] OR "ratio s"[All Fields] OR "ratioes"[All Fields] OR "ratios"[All Fields])) OR ("C-Reactive Protein"[MeSH Terms] OR ("C-Reactive Protein"[MeSH Terms] OR ("c reactive"[All Fields] AND "protein"[All Fields]) OR "C-Reactive Protein"[All Fields] OR "C-Reactive Protein"[All Fields]) OR ("C-Reactive Protein"[MeSH Terms] OR ("c reactive"[All Fields] AND "protein"[All Fields]) OR "C-Reactive Protein"[All Fields] OR "C-Reactive Protein"[All Fields]) OR ("C-Reactive Protein"[MeSH Terms] OR ("c reactive"[All Fields] AND "protein"[All Fields]) OR "C-Reactive Protein"[All Fields] OR "hscrp"[All Fields]) OR ("C-Reactive Protein"[MeSH Terms] OR ("c reactive"[All Fields] AND "protein"[All Fields]) OR "C-Reactive Protein"[All Fields] OR "high sensitivity c reactive protein"[All Fields]) OR ("C-Reactive Protein"[MeSH Terms] OR ("c reactive"[All Fields] AND "protein"[All Fields]) OR "C-Reactive Protein"[All Fields] OR "high sensitivity c reactive protein"[All Fields]) OR ("C-Reactive Protein"[MeSH Terms] OR ("c reactive"[All Fields] AND "protein"[All Fields]) OR "C-Reactive Protein"[All Fields] OR ("hs"[All Fields] AND "crp"[All Fields]) OR "hs crp"[All Fields]) OR ("curr res psychol"[Journal] OR "crp"[All Fields])) | 149,136 |
| 33 | ((("neutrophil s"[All Fields] OR "neutrophiles"[All Fields] OR "neutrophilic"[All Fields] OR "neutrophillic"[All Fields] OR "neutrophils"[MeSH Terms] OR "neutrophils"[All Fields] OR "neutrophil"[All Fields] OR "neutrophile"[All Fields]) AND ("lymphocytes"[MeSH Terms] OR "lymphocytes"[All Fields] OR "lymphocyte count"[MeSH Terms] OR ("lymphocyte"[All Fields] AND "count"[All Fields]) OR "lymphocyte count"[All Fields] OR "lymphocyte"[All Fields] OR "lymphocytic"[All Fields] OR "lymphocyts"[All Fields]) AND ("ratio"[All Fields] OR "ratio s"[All Fields] OR "ratioes"[All Fields] OR "ratios"[All Fields])) OR "NLR"[All Fields] OR "PLR"[All Fields] OR (("blood platelets"[MeSH Terms] OR ("blood"[All Fields] AND "platelets"[All Fields]) OR "blood platelets"[All Fields] OR "platelet"[All Fields] OR "platelets"[All Fields] OR "platelet s"[All Fields] OR "plateletes"[All Fields]) AND ("lymphocytes"[MeSH Terms] OR "lymphocytes"[All Fields] OR "lymphocyte count"[MeSH Terms] OR ("lymphocyte"[All Fields] AND "count"[All Fields]) OR "lymphocyte count"[All Fields] OR "lymphocyte"[All Fields] OR "lymphocytic"[All Fields] OR "lymphocyts"[All Fields]) AND ("ratio"[All Fields] OR "ratio s"[All Fields] OR "ratioes"[All Fields] OR "ratios"[All Fields])) OR "GPS"[All Fields] OR (("glasgow"[All Fields] OR "glasgow s"[All Fields]) AND ("prognostic"[All Fields] OR "prognostical"[All Fields] OR "prognostically"[All Fields] OR "prognosticate"[All Fields] OR "prognosticated"[All Fields] OR "prognosticates"[All Fields] OR "prognosticating"[All Fields] OR "prognostication"[All Fields] OR "prognostications"[All Fields] OR "prognosticator"[All Fields] OR "prognosticators"[All Fields] OR "prognostics"[All Fields]) AND ("score"[All Fields] OR "score s"[All Fields] OR "scored"[All Fields] OR "scores"[All Fields] OR "scoring"[All Fields] OR "scorings"[All Fields])) OR "LMR"[All Fields] OR (("lymphocytes"[MeSH Terms] OR "lymphocytes"[All Fields] OR "lymphocyte count"[MeSH Terms] OR ("lymphocyte"[All Fields] AND "count"[All Fields]) OR "lymphocyte count"[All Fields] OR "lymphocyte"[All Fields] OR "lymphocytic"[All Fields] OR "lymphocyts"[All Fields]) AND ("monocyte s"[All Fields] OR "monocytes"[MeSH Terms] OR "monocytes"[All Fields] OR "monocyte"[All Fields] OR "monocytic"[All Fields]) AND ("ratio"[All Fields] OR "ratio s"[All Fields] OR "ratioes"[All Fields] OR "ratios"[All Fields])) OR ("C-Reactive Protein"[MeSH Terms] OR ("C-Reactive Protein"[MeSH Terms] OR ("c reactive"[All Fields] AND "protein"[All Fields]) OR "C-Reactive Protein"[All Fields] OR "C-Reactive Protein"[All Fields]) OR ("C-Reactive Protein"[MeSH Terms] OR ("c reactive"[All Fields] AND "protein"[All Fields]) OR "C-Reactive Protein"[All Fields] OR "C-Reactive Protein"[All Fields]) OR ("C-Reactive Protein"[MeSH Terms] OR ("c reactive"[All Fields] AND "protein"[All Fields]) OR "C-Reactive Protein"[All Fields] OR "hscrp"[All Fields]) OR ("C-Reactive Protein"[MeSH Terms] OR ("c reactive"[All Fields] AND "protein"[All Fields]) OR "C-Reactive Protein"[All Fields] OR "high sensitivity c reactive protein"[All Fields]) OR ("C-Reactive Protein"[MeSH Terms] OR ("c reactive"[All Fields] AND "protein"[All Fields]) OR "C-Reactive Protein"[All Fields] OR "high sensitivity c reactive protein"[All Fields]) OR ("C-Reactive Protein"[MeSH Terms] OR ("c reactive"[All Fields] AND "protein"[All Fields]) OR "C-Reactive Protein"[All Fields] OR ("hs"[All Fields] AND "crp"[All Fields]) OR "hs crp"[All Fields]) OR ("curr res psychol"[Journal] OR "crp"[All Fields]))) AND ("Osteosarcoma"[MeSH Terms] OR ("Osteosarcoma"[MeSH Terms] OR "Osteosarcoma"[All Fields] OR "osteosarcomas"[All Fields]) OR ("Osteosarcoma"[MeSH Terms] OR "Osteosarcoma"[All Fields] OR "osteosarcomas"[All Fields]) OR ("Osteosarcoma"[MeSH Terms] OR "Osteosarcoma"[All Fields] OR ("Osteosarcoma"[All Fields] AND "tumor"[All Fields]) OR "osteosarcoma tumor"[All Fields]) OR ("Osteosarcoma"[MeSH Terms] OR "Osteosarcoma"[All Fields] OR ("Osteosarcoma"[All Fields] AND "tumors"[All Fields]) OR "osteosarcoma tumors"[All Fields]) OR ("Osteosarcoma"[MeSH Terms] OR "Osteosarcoma"[All Fields] OR ("tumor"[All Fields] AND "Osteosarcoma"[All Fields]) OR "tumor osteosarcoma"[All Fields]) OR ("Osteosarcoma"[MeSH Terms] OR "Osteosarcoma"[All Fields] OR ("tumors"[All Fields] AND "Osteosarcoma"[All Fields]) OR "tumors osteosarcoma"[All Fields]) OR ("Osteosarcoma"[MeSH Terms] OR "Osteosarcoma"[All Fields] OR ("sarcoma"[All Fields] AND "osteogenic"[All Fields]) OR "sarcoma osteogenic"[All Fields]) OR ("Osteosarcoma"[MeSH Terms] OR "Osteosarcoma"[All Fields] OR ("osteogenic"[All Fields] AND "sarcomas"[All Fields]) OR "osteogenic sarcomas"[All Fields]) OR ("Osteosarcoma"[MeSH Terms] OR "Osteosarcoma"[All Fields] OR ("sarcomas"[All Fields] AND "osteogenic"[All Fields]) OR "sarcomas osteogenic"[All Fields]) OR ("Osteosarcoma"[MeSH Terms] OR "Osteosarcoma"[All Fields] OR ("osteogenic"[All Fields] AND "sarcoma"[All Fields]) OR "osteogenic sarcoma"[All Fields])) | 59 |
