# Supplementary material for: Systemic Inflammatory Markers for Predicting Overall Survival in Patients with Osteosarcoma: A Systematic Review and Meta-Analysis
Source: Mediators Inflamm. 2021 Oct 21;2021:3456629. doi: 10.1155/2021/3456629 (PMC8553478; doi:10.1155/2021/3456629)
Supplement: Supplementary Materials — Supplementary File 1: search strategy for Medline database. Supplementary File 2: sensitivity analysis of the association of NLR, CRP, LMR, GRS, and PLR levels with the OS of patients with osteosarcoma. Supplementary File 3: Begg's funnel plot of the association of NLR, CRP, and GPS levels with the OS of patients with osteosarcoma. [file 3456629.f1.zip › 5.Supplementary Materials Figure.docx]

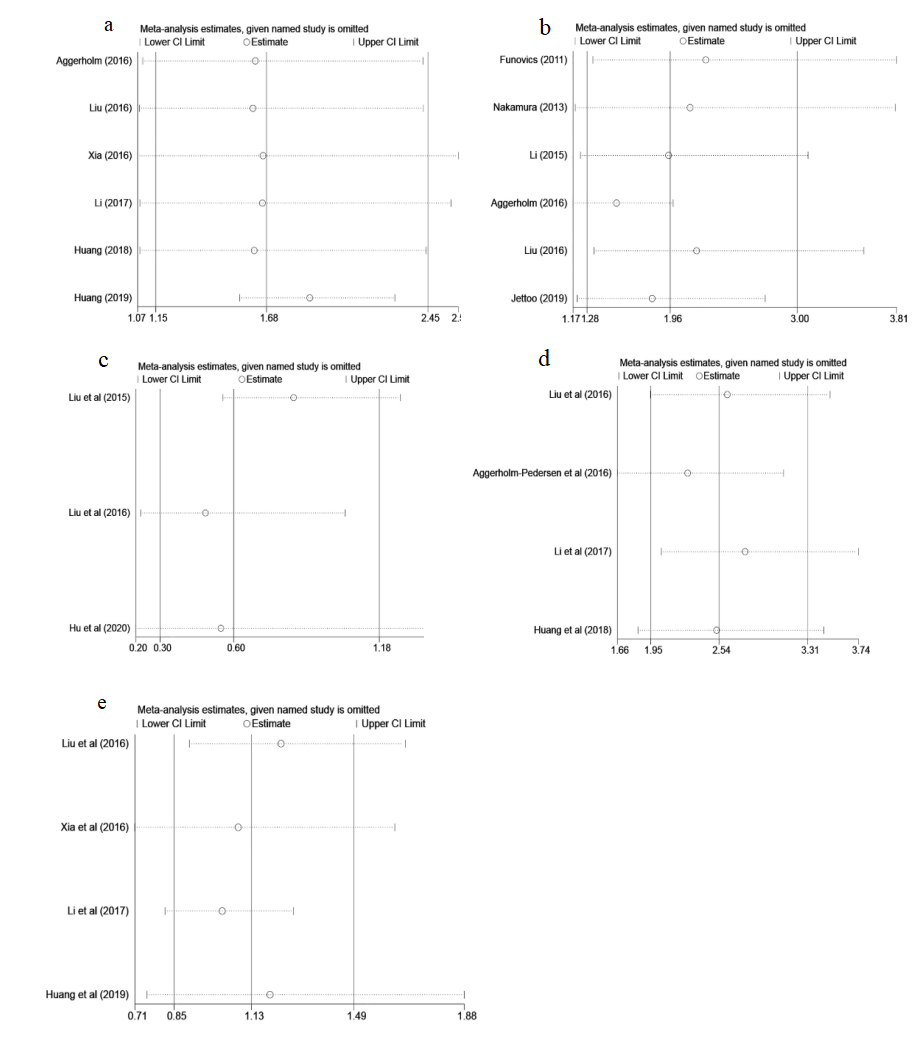


**Supplementary file 2**. Sensitivity analysis of the association of NLR(a), CRP(b), LMR(c), GRS(d), PLR(e) levels with the OS of patients with osteosarcoma.


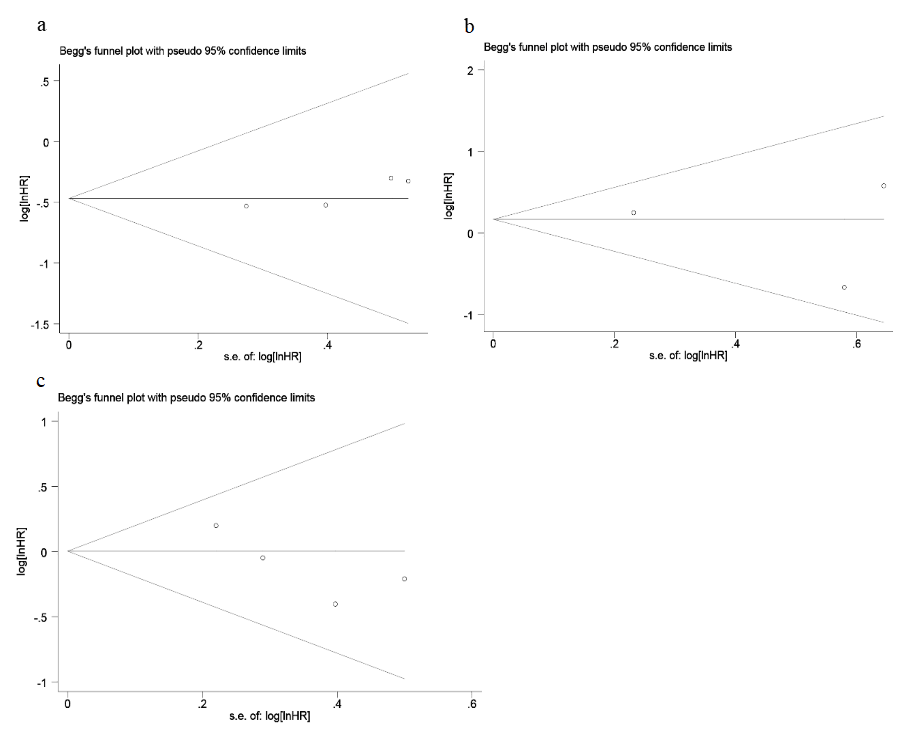


**Supplementary file 3**. Begg’s funnel plot of the association of NLR (a), CRP (b) and GPS (c)levels with the OS of patients with osteosarcoma.
